# Histocompatibility Antigen, Class I, G (HLA-G)’s Role during Pregnancy and Parturition: A Systematic Review of the Literature

**DOI:** 10.3390/life11101061

**Published:** 2021-10-09

**Authors:** Ourlad Alzeus G. Tantengco, Lauren Richardson, Alan Lee, Ananthkumar Kammala, Mariana de Castro Silva, Hend Shahin, Samantha Sheller-Miller, Ramkumar Menon

**Affiliations:** 1 Division of Basic and Translational Research, Department of Obstetrics & Gynecology, The University of Texas Medical Branch at Galveston, Galveston, TX 77551, USA; outanten@UTMB.EDU (O.A.G.T.); lestaffo@utmb.edu (L.R.); aljlee@UTMB.EDU (A.L.); ankammal@UTMB.EDU (A.K.); mqgmarianaa@gmail.com (M.d.C.S.); heshahin@utmb.edu (H.S.); sashelle@UTMB.EDU (S.S.-M.); 2 Department of Biochemistry and Molecular Biology, College of Medicine, University of the Philippines Manila, Manila 1101, Philippines; 3 Department of Pathology, Botucatu Medical School, Universidade Estadual Paulista, UNESP, Botucatu 18618-687, São Paulo, Brazil

**Keywords:** fetal membranes, placenta, antigen, immune tolerance, pregnancy

## Abstract

Introduction: Immune homeostasis of the intrauterine cavity is vital for pregnancy maintenance. At term or preterm, fetal and maternal tissue inflammation contributes to the onset of labor. Though multiple immune-modulating molecules are known, human leukocyte antigen (HLA)-G is unique to gestational tissues and contributes to maternal–fetal immune tolerance. Several reports on HLA-G’s role exist; however, ambiguity exists regarding its functional contributions during pregnancy and parturition. To fill these knowledge gaps, a systematic review (SR) of the literature was conducted to better understand the expression, localization, function, and regulation of HLA-G during pregnancy and parturition. Methods: A SR of the literature on HLA-G expression and function reported in reproductive tissues during pregnancy, published between 1976–2020 in English, using three electronic databases (SCOPE, Medline, and ClinicalTrials.gov) was conducted. The selection of studies, data extraction, and quality assessment were performed in duplicate by two independent reviewers. Manuscripts were separated into three categories: (1) expression and localization of HLA-G, (2) regulators of HLA-G, and (3) the mechanistic roles of HAL-G. Data were extracted, analyzed, and summarized. Results: The literature search yielded 2554 citations, 117 of which were selected for full-text evaluation, and 115 were included for the final review based on our inclusion/exclusion criteria. HLA-G expression and function were mostly studied in placental tissue and/or cells and peripheral blood immune cells, while only 13% of the studies reported data on amniotic fluid/cord blood and fetal membranes. Measurements of soluble and membranous HLA-G were determined mostly by RNA-based methods and protein by immunostaining, Western blot, or flow cytometric analyses. HLA-G was reported to regulate inflammation and inhibit immune-cell-mediated cytotoxicity and trophoblast invasion. Clinically, downregulation of HLA-G is reported to be associated with poor placentation in preeclampsia and immune cell infiltration during ascending infection. Conclusions: This SR identified several reports supporting the hypothesized role of immune regulation in gestational tissues during pregnancy. A lack of rigor and reproducibility in the experimental approaches and models in several reports make it difficult to fully elucidate the mechanisms of action of HLA-G in immune tolerance during pregnancy.

## 1. Introduction

Human pregnancy represents a unique immunological physiology where two distinct immune systems coexist to achieve successful gestation. The mystery of this immune state was identified almost 70 years ago and compared to an allogeneic conceptus with maternal and paternal antigens [1]. Many theories have since been proposed to explain maternal–fetal immune tolerance during pregnancy. There are currently five theories, each with strengths and weaknesses [2], including: placenta as a mechanical barrier [3], systemic suppression of the immune system [4], TH2 immune shift [5], lack of classical human leukocyte antigen (HLA) expression and expression of HLA-G [6,7,8], and local immune suppression [9,10]. However, the functional role and mechanistic contribution of HLA-G during pregnancy and parturition as an immune regulator is not yet known. 

HLA-G, one of the many HLAs, is a member of the non-classical major histocompatibility complex (MHC) class 1 molecules. HLA-G was first discovered in 1982 and received its final name in 1990 [11,12]. It is unique in that it is physiologically expressed in the placenta, specifically in extravillous trophoblasts; the immune-privileged cornea; some thymic epithelial cells; and pancreatic islet cells [6,13,14,15,16]. Pathologically, HLA-G expression is found in malignancies such as melanoma, where it is implicated in oncologic immune tolerance [17]. Functionally, HLA-G interaction with natural killer (NK) cells is associated with its inactivation, an important mechanism needed to maintain immune tolerance at the maternal–fetal interface [18,19,20,21]. At the molecular level, HLA-G is currently known to exist in seven different isoforms: four membrane-bound (HLA-G1, G2, G3, and G4) and three soluble (sHLA-G5, G6, and G7) [22,23,24,25]. HLA-G is dominantly expressed as a membrane-bound molecule on the surface of cells but can also be found in its soluble form in body fluids, such as within peripheral blood or amniotic fluid [26]. sHLA-G is generated either by shedding or cleavage of the membrane-bound form which is then secreted as the soluble form. Both forms of HLA-G are known to have anti-inflammatory and immunosuppressive properties as well as function similarly [27,28]. The regulation of HLA-G is different from other HLA molecules and is suspected to utilize upstream enhancers as regulatory elements; however, its precise molecular regulation remains unknown. Additionally, there is low polymorphism in the HLA-G gene and the HLA-G promoter does not interact with canonical inflammatory pathways such as NF-κB or IFN-γ [29].

In pregnancy, trophoblasts do not express the classical antigen-presenting HLA-A and HLA-B molecules, and instead only express HLA-C, HLA-G, and HLA-E [30]. A review by Ferreira et al. identified several possible mechanisms of HLA-G immunomodulation, highlighting its ability to: direct NK cell inhibition, NK cell reprogramming via endosomal signaling, trogocytosis, and macrophage modulation; control the release of IL-6 and IL-8; and direct T cell inhibition during pregnancy [29]. The unique expression pattern of HLA-G on extravillous trophoblasts positioned at the placental maternal–fetal boundary combined with the immunomodulating functions of HLA-G suggest it plays a role as a key immunomodulator at the maternal–fetal interface. sHLA-G, a soluble molecule, can be detected in pregnant women in all three trimesters. During the first trimester, this molecule plays an important role during the implantation process as it can regulate the activity of immune cells, such as NKs and cytotoxic T lymphocytes in the decidua, ensuring antigen tolerance [31]. The maternal sHLA-G concentration in patients who delivered at term by c-section showed that its levels continue to increase as labor progresses. The different concentrations of sHLA-G observed suggests that it plays a role in labor, likely regulating the immune homeostasis of the reproductive tract [32]. In preterm birth, the placental cell surface expression of HLA-G is higher. The mechanism associating HLA-G and pregnancy outcomes is not completely understood, but some studies are reporting that it can be related to gene expression and the interactions between maternal immune cells [33].

Ambiguity still exists regarding the immunomodulatory role of HLA-G during pregnancy and parturition. This can be partly attributed to HLA-G’s tissue-specific and local-environment-dependent functional differences as well as the distinct regulatory mechanisms that control its function. A better understanding of tissue-specific expression and functional changes, if any, under specific endogenous and exogenous conditions is required to better understand the contributions of HLA-G during pregnancy and parturition. Therefore, the objective of this review is to determine the localization, differential expression, immune and non-immune functions, regulators, and regulatory mechanisms of HLA-G functions in reproductive tissues during pregnancy, as well as in term and preterm parturition.

## 2. Methods 

To meet the requirements of the MOOSE group [34] and PRISMA statement [35], this systematic review of the literature was registered in PROSPERO (CRD42020214080).

### 2.1. Search Strategy

A systematic review of the literature published from 1976–2020 in English was collected from three databases: Ovid Medline, SCOPE, and CINAHL, with the assistance of a librarian team at the University of Texas Medical Branch at Galveston, TX, USA. A search strategy was developed to study HLA-G’s expression, function, and mechanistic role at the maternal–fetal interface (i.e., placenta and fetal membranes) during pregnancy (Table S1). 

### 2.2. Selection Criteria

Types of studies: The focus of this review was restricted primarily to the role of soluble and membrane-bound HLA-G in human pregnancy. Original research studies were selected, which investigated HLA-G at the maternal–fetal interfaces (i.e., placenta and fetal membranes) during pregnancy and parturition. Studies were excluded if they were not related to HLA-G, not related to pregnancy or parturition, focused on animal studies or assisted reproductive technologies (ART), were review articles, had a poor quality score, or the full text was not available. Articles were included if they reported samples from subjects at full-term gestation (≥37 weeks) who were either in labor or not in labor at the time of sample collection. Studies were also included if the results were solely related to patients with preterm labor and delivery (both spontaneous and induced), chorioamnionitis, preeclampsia, or studies that utilized cell lines from gestational tissues irrespective of maternal age, fetal gender, sociodemographic and other clinical factors, geographic location, ethnicity, or race. 

Types of outcome measures: Three types of outcome measurements reported were extracted for data analysis: (1) localization and expression changes associated with HLA-G at the maternal–fetal interface, (2) regulators of HLA-G, and (3) the mechanistic roles of HAL-G, during pregnancy and term or preterm parturition in humans. 

### 2.3. Data Collection and Analysis

Selection of studies: All citations retrieved through our search strategy were reviewed through online platforms. A.L. and L.R. independently screened the title and abstracts of the articles. Titles and abstracts that were not related to HLA-G, did not fit our inclusion criteria, or were review articles were removed and duplicates were excluded using Microsoft Access. Studies that fulfilled the selection criteria were included for full-text review and data extraction. 

Data extraction: Data extraction was conducted through Microsoft Access to collect the following information: authors, year of publication, article title, the role of HLA-G in said article, assay type used to determine HLA-G expression or function, and gestational tissue studied. The authors of the present study (A.L., L.R., S.S-M., A.K., O.T., H.S. and M.C.S.) independently extracted data from included articles, compared their findings, resolved disagreements through discussion, and produced a single, final form for each included study, organized through Microsoft Excel. 

Quality assessment: This systematic review utilized a validated quality assessment table and study methods as previously described [36,37,38]. Each article was analyze, and a score was given, ranking them as poor, acceptable, or good quality.

### 2.4. Data Synthesis

Based on our inclusion/exclusion criteria as described above, data that reported HLA-G’s role at the maternal–fetal interface during pregnancy and term or preterm parturition in humans were gathered. Based on this data, we determined the functions of HLA-G in each of these tissues and documented the localization, expression, regulation, and function of HLA-G along with knowledge gaps that have yet to be filled. 

## 3. Results

**Figure 1:**
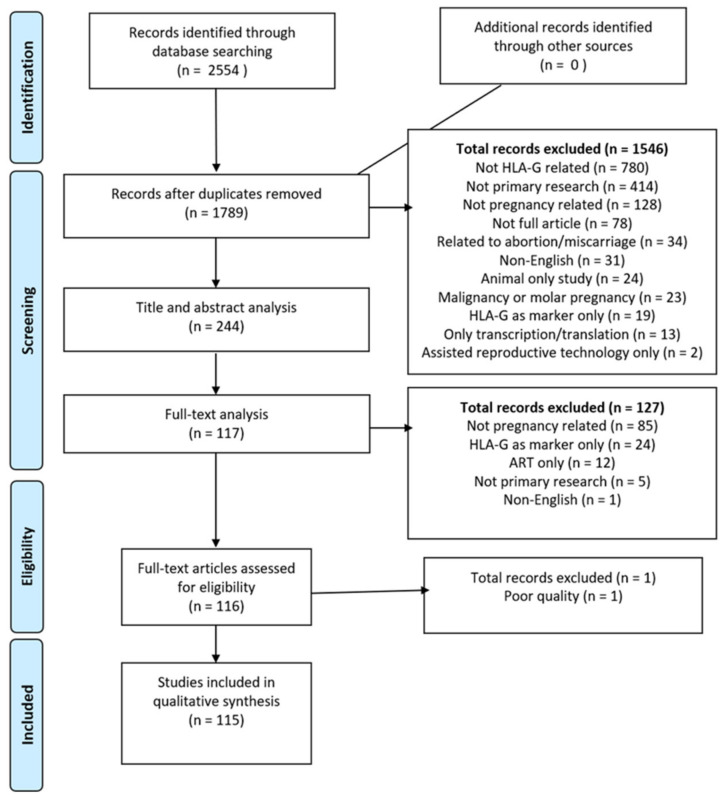
PRISMA flowchart. (PRISMA flowchart documenting the HLA-G systematic review search strategy).

**Figure 2:**
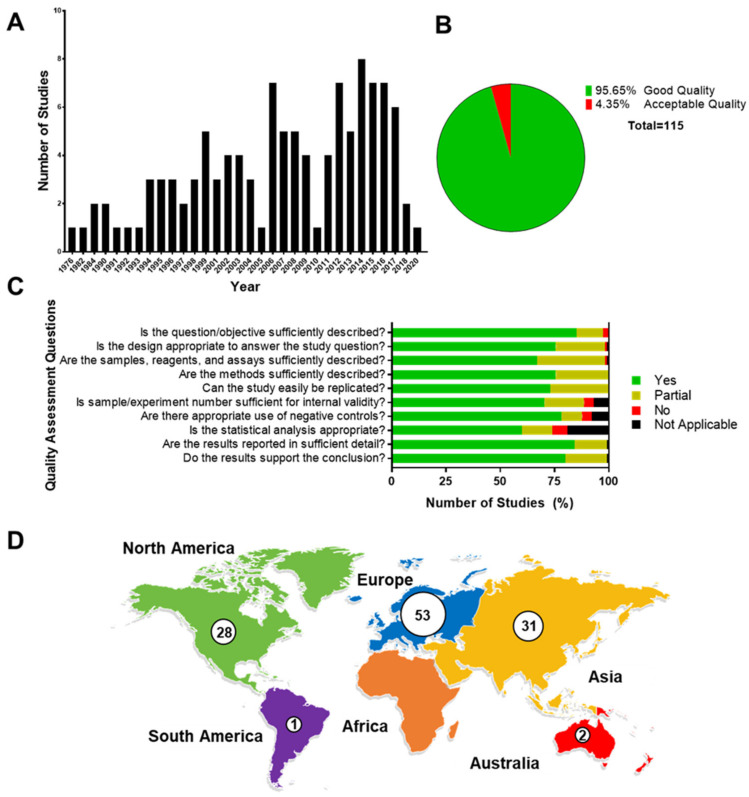
Systematic review quality assessment. (**A**) Annual distribution of published reports in HLA-G included in our systematic review. (**B**) Number of studies from our systematic review that scored “good quality” or “acceptable quality”. No papers were scored “poor quality”. (**C**) Quality assessment questions in studies included in the systematic review. Data presented as 100% stacked bars; figures in the stacks represent the number of studies. (**D**) Geographical distribution of published studies on HLA-G included in this systematic review.

The search within the three databases for reports published in English between 1960–2020 yielded 2554 citations. After removing duplicates 1789 remained. After screening citations by title and abstract, 244 articles remained. After the full-text review, 115 studies were included for the final data extraction and analysis (Figure 1; Table S1). In our chosen timeframe the first HLA-G article was published in 1976 and research reports reached a peak in 2014 (Figure 2A). Unfortunately, many unknowns still surround HLA-G’s expression, function, and regulation. There has been a decrease in HLA-G-related research reports in the past two years (Figure 2A).

### 3.1. Quality Assessment

One hundred and thirty-two (95%) manuscripts were graded as good quality, six (4%) were graded as acceptable quality, and one was graded as poor quality and was excluded from the study (Figure 2B). Good-quality articles provided an adequate description of sample ascertainment, study objectives, detailed reports on materials and methods, assay and analytical strategies, and an adequate description of data. Overall, manuscripts that received lower scores were predominantly lacking biological replicates, experimental controls, or proper statistical analysis (Figure 2C).

### 3.2. Main Characteristics of Studies

**Figure 3:**
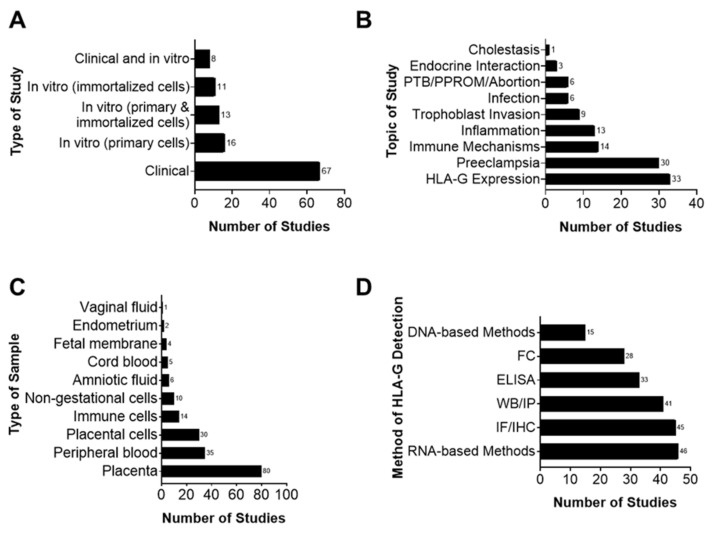
Main findings of HLA-G systematic review. (**A**) Type of study. Most studies included in our systematic review were clinical studies. (**B**) Topic of study. Most HLA-G articles studied histological and cellular expression as well as the localization of HLA-G and its role in the pathophysiology of preeclampsia. (**C**) The most commonly used tissue/sample for HLA-G studies is the placenta, followed by peripheral blood, placental cells, and immune cells. (**D**) The most common method used to detect HLA-G was RNA-based methods such as reverse transcriptase polymerase chain reaction and Northern blot analysis, followed by immunohistochemical staining of HLA-G protein.

Characteristics of the main findings are summarized in Table S1. Studies were conducted in labs with diverse research backgrounds, predominantly from Europe (60%), Asia (35%, limited to China), and North America (32%) (Figure 2D). Manuscripts included in this report investigated various biological processes involved in pregnancy and term or preterm parturition. While HLA-G was predominantly investigated in a clinical setting (50 studies), several in vitro cell culture experiments were also conducted (Figure 3A). These manuscripts primarily focused on HLA-G expression (33 studies) and its role in preeclampsia (30 studies), but also looked at its role in immune cell activation, regulation of inflammation, and trophoblast invasion (Figure 3B). 

### 3.3. Methods Used to Detect HLA-G Expression and Interactions

Studies in this review included the use of tissue and cells from fetal (i.e., fetal membranes, cord blood, amniotic fluid, and placenta) and maternal compartments (i.e., vaginal fluid, endometrium, immune cells, and peripheral blood), as well as experiments supplemented with in vivo animal models (Figure 3C). Note that the placenta was investigated the most (80 studies), while the fetal membranes were only mentioned four times (Figure 3C). The role of HLA-G and its interactions during pregnancy were analyzed by a variety of in vitro methods. The presence of HLA-G in its membrane-bound or soluble form was determined by RNA- or DNA-based methods and then confirmed with immunostaining or Western blot analysis at the protein level in the tissues mentioned above (Figure 3D). No knockdown or inhibitor studies were conducted to confirm the functional roles of HLA-G in these tissues.

### 3.4. Main Findings

Most published reports on soluble or membrane-bound HLA-G were clinical studies that used human samples (67 studies). There were also in vitro studies that used primary (16 studies) and immortalized (11 studies) human cells to investigate the role of HLA-G in pregnancy and parturition. Some of the HLA-G studies utilized maternal tissues and samples, i.e., maternal blood, while 14 of the HLA-G studies were on maternal–fetal immune cells. However, besides the placenta, very few studies focused on fetal tissues and samples such as amniotic fluid, cord blood, and fetal membranes. Other maternal tissues involved in pregnancy and parturition, such as the uterus and the cervix, were also not studied.

### 3.5. The Role of HLA-G in Placentation

**Figure 4:**
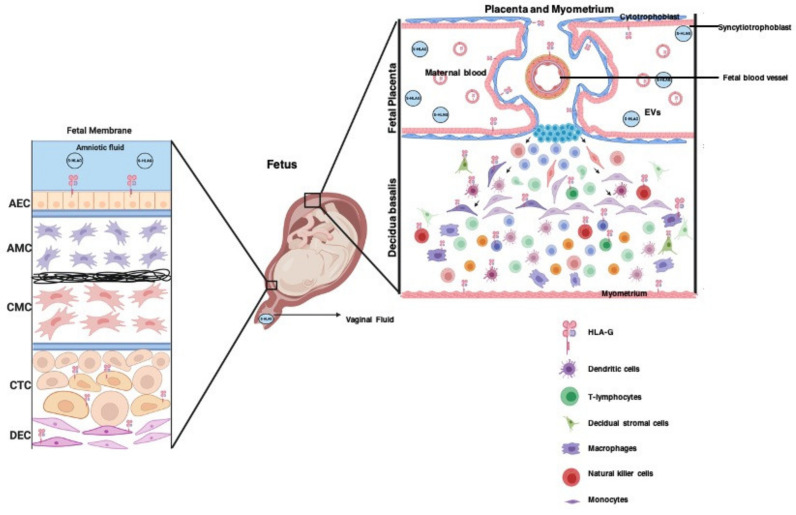
The localization of membrane-bound and soluble HLA-G proteins in the maternal–fetal microenvironment. Soluble HLA-G (sHLA-G) is present in vaginal fluid, maternal blood, cord blood, and amniotic fluid. Membrane-bound HLA-G is expressed in the fetal tissues, including the fetal membrane (AEC, CTC, and decidua cells) and fetal blood vessels. It is also expressed in the placenta, particularly in the cytotrophoblasts, syncytiotrophoblasts, and maternal immune cells (dendritic cells, lymphocytes, macrophages, monocytes, and natural killer cells), decidual stromal cells, and myometrial cells. Extracellular vesicles from the placenta also express HLA-G. However, it is still unknown whether these HLA-G-positive EVs serve as a paracrine-signaling mechanism to facilitate the effect of HLA-G in neighboring cells.

Most studies on reproductive tissues were conducted in placental tissues and cells. Both membrane-bound and soluble isoforms of HLA-G were found to be localized and expressed in placental trophoblasts (i.e., cytotrophoblasts and syncytiotrophoblasts), placental immune cells (i.e., macrophages, natural killer (NK) cells, and T lymphocytes), and fetal membranes (Figure 4). mHLA-G expression was found to be higher in these cells during the first trimester than in the last trimester of pregnancy [6]. Moreover, mHLA-G was reported to prevent immune-cell-mediated cytotoxicity in extravillous trophoblasts (EVT). Actively migrating EVT express mHLA-G [39]; however, a subset of EVT that do not express mHLA-G were found to show signs of necrosis in human placentas [40]. sHLA-G5 was also shown to promote trophoblast invasion through binding to its receptors on cells (KIR2DL4 and LILRB1), which increases urokinase and matrix metallopeptidase expression, and activates the ERK signaling pathway [39,41,42]. The chorion plate mesenchymal stem cells have also been shown to express mHLA-G, promoting immunosuppression and trophoblast invasion [43]. These studies indicate the potential role of membrane-bound and soluble HLA-G in trophoblast viability, invasion, differentiation, and immunologic function at the placental maternal–fetal interface. 

HLA-G was also reported in decidualization during pregnancy. Anti-inflammatory cytokines such as IL-10, IFN-y, and progesterone increased the expression of HLA-G in decidual stromal cells. Progestins also increased HLA-G transcription along with decidualization of human endometrial stromal cells [44]. These studies indicate the role of HLA-G in placentation and decidualization. 

### 3.6. The Role of HLA-G in Promoting Maternal–Fetal Tolerance in Pregnancy

To promote immune tolerance at the maternal–fetal interface, HLA-G exhibits a variety of anti-inflammatory properties in the decidua and placenta. Within the decidua, sHLA-G was shown to (1) reduce TNF-α and IFN-y production in decidual mononuclear cells [45], (2) increase IL-12 and IL-4 production in macrophages and CD45+ cells, respectively [46], and (3) inhibit pro-inflammatory cytokine production after stimulation with LPS or peptidoglycan in sHLA-G5-expressing decidual dendritic cells (DCs) [47]. Similarly, sHLA-G contributed to the reduction in pro-inflammatory cytokine production in decidual large granular lymphocytes [48], induced a Th2 cytokine profile state in decidual mononuclear cells and peripheral blood mononuclear cells [49], and prevented the maturation of DCs during infection [47]. Overall, these studies highlight the ability of sHLA-G and HLA-G to modulate decidual immune cells by reducing pro-inflammatory and increasing anti-inflammatory cytokine production, thus maintaining maternal–fetal tolerance during pregnancy. 

The expression of HLA-G in peripheral blood and placental immune cells also plays a role in immunomodulation and stopping the maternal immune system from attacking the developing fetus. In maternal peripheral blood, DCs (CD14+, SIGN+) express more HLA-G than DCs (CD14+, SIGN-) in placental immune cells; DCs (CD14+, SIGN+) were also shown to induce immunosuppressive Treg cells [50]. Furthermore, peripheral-blood-granulocytic-myeloid-derived suppressor cell activity was increased by HLA-G immunoglobulin-like transcript (ILT) 2 and ILT4 signaling [51]. These studies reference HLA-G’s ability to modulate immune cells within the maternal peripheral blood.

This systematic review also identified studies that describe HLA-G’s ability to inhibit the cytotoxic activity of immune cells in the decidua and placenta. HLA-G preserves the activation/inhibition balance in decidua NK during the first trimester of pregnancy [52]. Interaction of decidual NK cells with HLA-G-expressing EVT leads to the acquisition of HLA-G by decidual NKs through trogocytosis. This acquisition of HLA-G inhibits the cytotoxicity of decidual NKs [18]. In another study, Tilburgs et al. showed that placental NK cells, which are more exposed to fetal cells and antigens, expressed higher levels of HLA-G compared to peripheral NK cells, which did not express HLA-G [18,29]. Downregulation of HLA-G in placental JEG-3 cells increased NK-mediated cell killing [53]. Knockdown of HLA-G diminished EVT cell resistance to NK-mediated cytotoxicity [54]. On the other hand, HLA-G-transfected LCL 721.221 HLA-null cells became resistant to decidual NK lysis. sHLA-G was also shown to induce apoptosis in decidual NK cells [55,56]. These reports highlight the role of HLA-G in maintaining cell viability and maternal–fetal tolerance at this critical interface [57].

### 3.7. The Role of HLA-G in the Pathophysiology of Pregnancy Complications

HLA-G also plays a role in the pathophysiology of pregnancy complications. Levels of sHLA-G were significantly lower in maternal serum and plasma of patients with preeclampsia (PE) [58,59,60,61]. Moreover, peripheral blood CD45+ HLA-G+ cells from PE patients expressed lower HLA-G levels than patients with a normal pregnancy [62]. This reduction in HLA-G level in PE patients may be due to the hypermethylation of HLA-G promoter regions [63]. miRNA also plays a role in the regulation of HLA-G expression. miR-148a, which upregulates HLA-G post-transcriptionally, was significantly decreased in PE patients. Placentas from PE patients expressed lower HLA-G mRNA and protein levels compared to patients with a normal pregnancy [64,65,66,67,68]. Moreover, oxidative stress, which is associated with PE, was shown to decrease the expression of HLA-G in placental tissues [69].

Several studies also investigated the expression of membrane-bound and soluble HLA-G in umbilical cord blood and its association with preeclampsia [26,43,70,71,72,73,74]; however, its expression was significantly lower than in maternal blood [72]. In contrast to the results in maternal blood, no association was found between HLA-G polymorphisms in the umbilical cord blood and PE and fetal growth restriction [70,73,74]. Previous studies from Biyik et al. and Marozio et al. reported that the first trimester maternal serum level of HLA-G was not associated with preeclampsia [75]. However, HLA-G was found to be associated with placental abruption and with overall pregnancy complications [60]. Several reports have shown that sHLA-G was significantly increased in the maternal blood of patients with pPROM, uncontrollable PTL, and PTB. sHLA-G levels can be used to predict the occurrence of preeclampsia and IUGR [58,76,77]. 

Both forms of HLA-G may also play a role in maternal and fetal infections during pregnancy. However, it is still unknown whether HLA-G plays a protective or pathologic role in maternal and fetal infections. sHLA-G levels were elevated in the maternal blood of patients with congenital cytomegalovirus infections [26]. Most studies on amniotic fluid looked at the expression of sHLA-G in the presence of infection. Maternal and fetal infections (i.e., cytomegalovirus infection and toxoplasmosis) increased the levels of sHLA-G in the amniotic fluid [26,78,79]. These studies reported the expression and regulation of HLA-G during PE, abortion, and infectious PTB, stressing its important role in these pregnancy complications. 

### 3.8. Extracellular Vesicles Package HLA-G during Pregnancy

Extracellular vesicles, such as microvesicles and exosomes, package protein cargo that plays a role in cell–cell communication during pregnancy. Previous studies have shown that HLA-G was also present in microparticles and exosomes released by placental cytotrophoblast cells [80]. However, HLA-G expression in exosomes weakened and/or disappeared when these cells were differentiated to STB. Orozco et al. also showed that DNA-associated microparticles from maternal plasma express HLA-G and placental alkaline phosphatases. DNA amounts per HLA-G+ MP increase in PE women, which might indicate dysfunctional extravillous cytotrophoblasts [81]. This shows that paracrine signaling via extracellular vesicles also plays a role in modifying the maternal–fetal immunological environment during pregnancy.

## 4. Discussion

A total of 115 clinical and experimental studies reporting HLA-G’s (including the soluble form of HLA-G) role during pregnancy and parturition were analyzed for this review. Human tissues and cells from the peripheral blood, uterus, vagina, and fetal origin (placenta, fetal membranes, umbilical vein endothelial cells, and umbilical cord blood) were used to evaluate HLA-G in these studies. The most reported functions of HLA-G in these studies include the regulation of inflammation, inhibition of immune cell-mediated cytotoxicity, trophoblast invasion and viability. Regulators of HLA-G in pregnancy-related tissues were also identified, and the role of HLA-G in different pregnancy complications including PE, PTB, and maternal and fetal infections during pregnancy is summarized. 

Studies have shown that HLA-G modulates immune cells by inhibiting NK cells and lymphocytes, as well as by reducing inflammation (i.e., pro-inflammatory cytokines) to prevent immune cell infiltration and to maintain immune homeostasis [52,54,57]. These are processes that are essential throughout gestation; however, it is not known whether HLA-G reduction alone at term contributes to immune intolerance. PE patients were reported to have low HLA-G levels in the placenta as well as within maternal and fetal blood, contributing to the disease [64,65,66,67], although it is still unclear if oxidative stress at term or during PE affects the expression of HLA-G, preventing it from modulating the immune environment. One of the major limitations of these studies is that a mechanistic model is yet to emerge to confirm causality based on associations. More mechanistic studies are needed to understand HLA-G’s role in regulating pro- and anti-inflammatory cytokine production within these tissues under different pregnancy conditions [46,47,49]. Furthermore, tissue- and cell-type-specific quantitative comparisons to assess HLA-G expression levels across feto-maternal tissues are currently lacking. In addition to modulating the immune system, HLA-G has been shown to prevent immune and placental cell cytotoxicity and induce migration/invasion in EVT, decidua, and chorion mesenchymal stem cells [40,41,43]. HLA-G’s ability to induce differentiation and migration should be investigated in the fetal membranes and cervical stroma, where mesenchymal cells contribute to collagen remodeling and tissue maintenance. HLA-G predominantly induces its functions through receptor–ligand interactions; however, sHLA-G and HLA-G can also be packaged into exosomes and induce endocrine signaling [80]. As it has previously been shown that exosomes play a major role in the induction of labor at term and preterm [82,83,84,85,86], further characterization of exosomes from different intrauterine tissues is needed to elucidate their role in maintaining maternal–fetal immune homeostasis. 

In general, it is thought that HLA-G is regulated through upstream enhancers and elements that differ from other HLA molecules; however, little is known about its regulation in pregnancy-related tissues. In this review we have identified regulators of HLA-G (i.e., progestins and IL-10) and how they could contribute to HLA-G expression during placentation and decidualization [87,88,89]. These types of studies should also be carried out in other intrauterine tissues to determine their effects. Clinically, due to the application of progestins to treat short cervix or women with a history of PTB, HLA-G levels after treatment should be monitored to evaluate the usefulness of progestins in cases of PE. PE is a potentially dangerous pregnancy complication characterized by hypertensive disorders and signs of organ damage [90,91]. HLA-G expression was significantly reduced in the placenta as well as within the maternal and fetal blood of PE patients [64,65,66,67], and was shown to promote dysfunction in trophoblast invasion and placental angiogenesis as well as promote trophoblast cell death [39,40,92]. Thus, identifying a treatment that could increase HLA-G levels in utero could be beneficial. Additionally, discrepancies in reports showing HLA-G polymorphisms as a useful biomarker for PE varied greatly depending on the tissue and isoforms analyzed, highlighting the need for further investigation using HLA-G as a diagnostic or prognostic biomarker, or as a therapeutic target in specific obstetric complications. 

Along with PE, HLA-G is associated with congenital infections [26,78,79]. The significant increase in sHLA-G in the amniotic fluid as well as the maternal and fetal blood may indicate a role of sHLA-G in the immune response to combat infections during pregnancy. It may also promote immunomodulation to prevent fetal loss during pregnancy. However, an excessive increase in sHLA-G may be detrimental, as excessive immunosuppression may favor the transmission of congenital infections during pregnancy. These studies also showed the potential use of sHLA-G as a potential biomarker for intraamniotic infections, although non-invasive sample collections (i.e., vaginal/cervical fluid swabs) should be carried out as amniocentesis carries its own risks.

## 5. Future Directions 

This systematic review has shown us that HLA-G is now emerging as a major regulator of maternal–fetal tolerance, placentation, and viability in reproductive tissues. However, studies that look primarily at HLA-G’s regulation or function in the fetal membranes, cervix, and uterus are few but should be carried out, as it could play major roles in immune cell infiltration, cervical ripening, and collagen remodeling. Further studies to delineate the expression and function of HLA-G at term, under oxidative stress, and PTL in reproductive tissues are needed to understand HLA-G’s immunoregulatory role and its potential as a target for therapeutic interventions in adverse pregnancy events. Filling these gaps of knowledge will help advance the field and contribute to our overall understanding of HLA-G. 

## Supplementary Materials

The following are available online at https://www.mdpi.com/article/10.3390/life11101061/s1, Table S1: Studies included in qualitative analysis.
