# Supplementary material for: Histocompatibility Antigen, Class I, G (HLA-G)’s Role during Pregnancy and Parturition: A Systematic Review of the Literature
Source: Life (Basel). 2021 Oct 9;11(10):1061. doi: 10.3390/life11101061 (PMC8537334; doi:10.3390/life11101061)
Supplement: Supplementary file 1 [file life-11-01061-s001.zip › life-1342756-supplementary.pdf]

Authors	Year	Type of Study	Type of Sample	Methods	Topic of Study	Localization of HLA-G	HLA-G role in pregnancy	HLA-G interactions in pregnancy
A. King et al.	1996	Clinical and in vitro	CL (BEWO, JEG-3) Placenta	FC, IHC, WB	HLA-G Expression	1st trimester placental EVT		
J. Kusanovic et al.	2009	Clinical	Amniotic Fluid	ELISA	Infection	Amniotic fluid	HLA-G elevated in PTB and intraamniotic infection. sHLA-G may play role in immune response	
P. Vianna et al.	2007	Clinical	PB	DNA PCR	Preeclampsia		14 base pair polymorphism is associated with PE	
S. Kovats et al.	1990	Clinical	CL (Bewo, JAR, JEG-3) Placenta	WB	HLA-G Expression	Placental villous cytotrophoblast		More HLA-G in 1st trimester than 3rd trimester, HLA-G is transcriptionally regulated
R. Apps et al.	2007	In vitro (primary cells)	CL (JEG-3) Placenta	WB	Gestation	Placental trophoblast		LILRB2 on decidual myelomonocytic cells preferentially bind dimeric HLA-G of trophoblast cells, co-culture of HLA-G+ cells increases IL-6 and IL-10
X. Zhou et al.	2012	In vitro (immortalized cells)	CL (HTR-8/Svneo) Placenta,	IHC	Preeclampsia			Oxidative stress decreases HLA-G in placentas with PE.
C. Aldrich et al.	2000	Clinical	CB, Placenta	dPCR	Preeclampsia		HLA-G 1597 delta C null mutation not associated with PE or growth restriction	
S. Djurisic et al.	2015	Clinical	PB, Placenta	FC, ELISA	HLA-G Expression		Placental NK cells express more KIR2DL4 peripheral blood NK. sHLA-G levels are directly related to KIR2DL4 levels. sHLA-G was significantly increased in aborted fetal tissue compared with peripheral blood	
S. Yie et al.	2008	Clinical	CL (JEG-3), PB, Placenta	RT rPCR	Preeclampsia		A single base-pair mutation in the HLA-G gene 3′-untranslated region is associated with reduced HLA-G levels and shorter HLA-G half-lives in PE.	
P. Jahan et al.	2014	Clinical	PB	dPCR	Preeclampsia		14 base pair polymorphism is not associated with preeclampsia	
T. Kanai et al.	2003	In vitro (primary and immortalized cells)	CL (CRL-1855), Placenta	N/A	Inflammation		sHLA-G reduces TNF-α and IFN-γ but does not change IL-4 in decidual mononuclear cells	
P. Sedlmayr et al.	2002	Clinical and in vitro	Placenta	IHC, RT rPCR	HLA-G Expression	Not present on placental decidua macrophage		
S. Rajagopalan et al.	2006	In vitro (primary and immortalized cells)	CL (221-HLA-Cw3, 293T, NKL, YTS), PB	ELISA, IHC, RT rPCR	Inflammation		sHLA-G induced secretion of proinflammatory and proangiogenic cytokines by resting NK cells. HLA-G signals via endosomal signaling.	
M. Chauhan et al.	2011	In vitro (primary and immortalized cells)	CL (HTR-8SV/Neo) PB, Placenta	IF, RT rPCR, WB	Trophoblast Invasion		ADM2 upregulates HLA-G at the maternal-fetal interface facilitating trophoblast invasion and migration via MAPK3/1 phosphorylation	
P. Hsu et al.	2012	Clinical and in vitro	PB, Placenta	FC	Preeclampsia	Decidual CD14(+)DC-SIGN(+)	CD14+, SIGN+ express more HLA-G than CD14, SIGN- cells; CD14+SIGN+ cells induce immune suppressive Treg cells	
N. Hara et al.	1996	Clinical	Placenta	IHC	Preeclampsia	Placental trophoblast	Poor staining of HLA-G on trophoblasts in PE patients	
M. O'Brien et al.	2001	Clinical	Placenta	RT rPCR, Sequencing	Preeclampsia		Polymorphisms associated with lower HLA-G3 transcript was observed in mild PE	
M. Kirszenbaum et al.	1994	Clinical	PB, Placenta	RT rPCR	HLA-G Expression	Maternal serum		
K. Yelavarthi et al.	1991	Clinical	Placenta	IHC, ISH	HLA-G Expression	1st trimester placental cytotrophoblast, 1st trimester villous trophoblast (not 3rd), chorionic membrane trophoblast membrane trophoblast		
S. Rokhafrooz et al.	2018	Clinical	PB	ELISA, PCR	Preeclampsia	Soluble HLA-G found in serum	sHLA-G levels are decreased in PE	HLA-G expression is regulated by elements in 5' URR and 3' UTR
J. Emmery et al.	2017	Clinical	PB, CB	dPCR, Sequencing	HLA-G Expression		14bp HLA-G deletion is associated with higher birthweight and elevated expression of HLA-G on the trophoblast.	
B. His et al.	1984	Clinical	Placenta	IHC	HLA-G Expression	Fetal amniochorion		
S. Basak et al.	2020	In vitro (primary and immortalized cells)	CL (JEG, HMEC-1, HTR-8/Svneo, PC3)	RT rPCR, WB	HLA-G Expression			Curcumin stimulates HLA-G in vitro
Y. Yang et al.	1995	In vitro (primary and immortalized cells)	CL (JEG-3, Jar), Placenta	FC, IHC, NB, WB	HLA-G Expression			TNF-alpha, beta, and gamma, and TGF-alpha moderately enhance HLA-G mRNA without protein level changes
M. Petroff et al.	2002	In vitro (primary and immortalized cells)	CL (U937) Placenta	FC, RT PCR	Immune Mechanism			Decidual macrophages express the HLA-G receptors ILT2 and ILT4
A. Wallace et al.	2015	Clinical	Decidua NK cells, Placenta	FC	HLA-G Expression		Decidual NK cells from pregnancies with elevated uterine artery index express less KIR2DL and LILRB1	
S. Lou et al.	2018	Clinical	CL (HTR-8/Svneo), Placenta	RT PCR, WB	Preeclampsia		PE is associated with lower levels of JHDM1D. JHDM1D regulates HLA-G expression.	

J. Cross et al.	1999	In vitro (immortalized cells)	CL (JAR, JEG-3)	IHC, NB, WB	Inflammation			HLA-G is the predominant MHC class I antigen produced by JEG-3 cells, levels are unaffected by IFN
R. Hackmon et al.	2017	In vitro (primary cells)	Placenta	IHC	Trophoblast Invasion	1st trimester placental EVT	HLA-G is expressed on the surface of actively migrating EVT	
K. Kronquist et al.	1984	Clinical	Amniotic fluid, PB	WB	HLA-G Expression	Amniotic fluid		
X. Wei et al.	1990	Clinical	Placenta	NB	HLA-G Expression	1st trimester placenta, 3rd trimester placental EVT		
W. Faulk et al.	1976	Clinical	Placenta	IF	HLA-G Expression	B2-microglobulin not identified in trophoblasts		
L. Sun et al.	2008	In vitro (primary cells)	CL (JEG-3)	RT PCR, WB	Immune Mechanism		Downregulation of HLA-G is associated with increased NK-mediated cell killing	
R. Marlin et al.	2012	Clinical	Placenta	FC	Immune Mechanism	Decidual NK cells, antigen presenting cells, and T lymphocytes	HLA-G plays a role in activation homeostasis of decidual NK cells	
A. Steinborn et al.	2007	Clinical	PB	ELISA	Preeclampsia	Maternal serum	sHLA-G levels are high in 1st trimester then decrease. In 2nd trimester decreased HLA-G levels are associated with PE or growth restriction. In 3rd trimester increased sHLA-G associated with PTL	
J. Choi et al.	2014	In vitro (primary cells)	CL (HTR-8/Svneo, WI-38), CB, Placenta	ELISA, FC, IHC, RT rPCR, Zygometry	Trophoblast Invasion		Chorion plate mesenchymal stem cells activate immunosuppression and trophoblast invasion via HLA-G	Placental chorion plate derived mesenchymal stem cell coculture with trophoblasts increase sHLA-G
M. Du et al.	2014	In vitro (primary cells)	Placenta	ELISA, FC, IHC, WB	Inflammation			Decidual Treg cells promote HLA-G expression on trophoblasts
F. Shido et al.	2006	In vitro (immortalized cells)	CL (JEG-3)	ELISA, FC, WB	Trophoblast Invasion			ERAP1 is involved in the regulation of surface HLA-G on EVT in the presence of LIF
A. Blaschitz et al.	1997	Clinical	CL (JAR, JEG-3), Placenta	IHC	HLA-G Expression	1st trimester villous cytotrophoblast stem cells and endovascular trophoblast		
R. Apps et al.	2011	Clinical and in vitro	PB, Placenta	FC, IHC, dPCR	HLA-G Expression		1st trimester decidual NK cells are not responsive to HLA-G	14 bp polymorphism does not affect surface levels of HLA-G
P. Hsu et al.	2014	Clinical	CL (JEG-3), PB	FC	Inflammation		Lower levels of peripheral blood CD4+HLAG+ cells in PE	CD45+ SIGN+ antigen presenting cells enhance HLA-G expression on CD4 cells in vitro. IL-10 augments expression of HLA-G in monocyte derived DC
X. Zhang et al.	2007	Clinical	PB, miscarriage	IHC, dPCR	Infection		HLA-G expression in CMV+ is higher in placental villi. Intrauterine HCMV infection at early pregnant stage is closely related to HLA-G expression at the maternal-fetal interface.	
T. Komatsu et al.	1998	Clinical	Uterus (Hysterectomy), Placenta	IHC, NB	HLA-G Expression	Endometrium		Progestins increase HLA-G transcription along with decidualization of human endometrial stromal cells
O. Devergne et al.	2001	In vitro (immortalized cells)	CL (BEWO, BJAB, BL41, CEM-T, COS7, IB4, HL-60, Jar, Jurkat, Molt-4, NC37, Ramos, U937), Placenta	ELISA, IHC, IP, WB	Infection			Epstein Barr Virus induced gene 3 is presented by HLA-G
H. Hutter et al.	1996	Clinical	Placenta	IHC	Trophoblast Invasion	Invading trophoblast (beta2-m macroglobulin), Chorion laeve (HLA-G)		
X. Zhu et al.	2012	Clinical	Placenta	RT rPCR), WB	Preeclampsia		Placental HLA-G is decreased in PE	
G. Colbern et al.	1994	Clinical	Placenta	Ribonuclease protection assay	Preeclampsia		HLA-G levels are reduced in PE trophoblasts	
J. Proll et al.	1999	Clinical	Placenta	IHC	HLA-G Expression	1st trimester extravillous trophoblasts		
A. Farina et al.	2011	Clinical	CVS, Placenta	RT rPCR	Preeclampsia		HLA-G is decreased in 1st trimester patients that progress to PE	
D. Veljkovic Vujaklija et al.	2013	In vitro (immortalized cells)	CL (K562), Decidua, PB, Placenta	ELISA, FC, IHC	Inflammation		Decidual CD56+ cells decrease granulysin expression and secretion when co-cultured with K562 HLA-G expressing cells	
F. Robert-Gangneux et al.	2011	Clinical	Amniotic fluid	ELISA	Infection	Amniotic fluid	sHLA-G increased in amniotic fluid in pregnancies with toxoplasmosis	
J. Prins et al.	2016	Clinical	PB, Placenta	RT rPCR	Preeclampsia		HLA-G mRNA higher in PE decidual lymphocytes and macrophages	
G. Amodio et al.	2013	Clinical	PB, Placenta	FC	HLA-G Expression	DC-10 cells in peripheral blood and DC-10 and CD4+ cells in decidua		
T. Hviid et al.	2004	Clinical	Placenta	IHC, Microarray	Preeclampsia		Less HLA-G mRNA in PE patients	HLA-G genotype not associated with protein quantity
A. Asako et al.	2016	In vitro (immortalized cells)	CL (HChEpC1b), Placenta	Microarray, WB	Trophoblast Invasion			miRNA-365 represses HLA-G expression under hypoxic conditions
E. Ivanova-Todorova et al.	2009	In vitro (primary cells)	Adipose tissue, Bone marrow, Placenta	ELISA, RT rPCR, WB	Endocrine Interaction	Decidua-derived mesenchymal stem cells		Progesterone upregulates HLA-G
T. Hviid et al.	2004	Clinical	PB	RT rPCR	PTB/PPROM/Abortion		14 bp positive genotype associated with increased gestational age adjusted birth weight and placental weight	
M. McMaster et al.	1998	Clinical	Amniotic fluid, Fetal membranes, Placenta	IHC, WB	HLA-G Expression	Amniotic fluid		

N. Kostlin et al.	2017	Clinical	PB, Placenta	FC	Immune Mechanism		Peripheral blood granulocytic myeloid derived suppressor cells activity are increased by HLA-G ILT2 and ILT4 signaling	
C. Menier et al.	2000	Clinical and in vitro	CL (M8, YT2C2-PR), Placenta	FC, WB	Immune Mechanism			Truncated HLA-G forms can inhibit NK cells
L. Lombardelli et al.	2013	In vitro (primary cells)	CL (Tetanus toxoid (TT)–specific T CLs), PB	ELISA	Inflammation		sHLA-G5 increases IL-12 production from macrophages and IL-4 from CD4+ cells in vitro	
B. Hsi et al.	1982	Clinical	Decidua cells, Fetal membranes, PB, Placenta	IHC	HLA-G Expression	amniochorion chorion and amniotic epithelium lack B2 macroglobulin		
K. Lim et al.	1997	Clinical	Placenta	RT rPCR	Preeclampsia		HLA-G is down regulated in PE	
O. Blanco et al.	2008	Clinical	Placenta	FC, IF, RT rPCR, WB	Inflammation	Decidual stromal cells	IL-10, IFNy, progesterone, and cAMP (not IL-2) increase the expression of HLA-G in decidual stromal cells	
L. Klitkou et al.	2015	Clinical	CB, PB	ELISA	HLA-G Expression	Cord blood, Maternal serum	sHLA-G in maternal blood decreases as gestation advances, sHLA-G higher in maternal blood than in umbilical blood at term	
M. Dahl et al.	2015	Clinical	PB, Placenta	ELISA, dPCR, Sequencing	HLA-G Expression			Increasing number of 14 bp inserts associated with increased sHLA-G at term
X. Zhang et al.	2014	Clinical	Placenta	RT rPCR, WB	Cholestasis		miR-148a down regulates HLA-G in intrahepatic cholestasis of pregnancy	
T. Kanai et al.	2001	Clinical	CL (HLA class I-deficient B-lymphoblast CL, 221 cells), PB, Placenta	ELISA	Inflammation		HLA-G induces a Th2 cytokine profile state in decidual mononuclear cells and peripheral blood mononuclear cells	
M. McMaster et al.	1995	In vitro (primary and immortalized cells)	CL (JEG-3), Placenta	IHC, NB	HLA-G Expression	Placental cytotrophoblast	HLA-G mRNA levels decrease as gestation progresses	
Y. Tang et al.	2015	Clinical	PB, Placenta	ELISA, IHC, Sequencing RT rPCR	Preeclampsia		HLA-G is hypermethylated in PE and HLA-G levels are decreased	
T. Nagamatsu et al.	2004	In vitro (primary cells)	Placenta	FC, RT rPCR, WB	HLA-G Expression			Hypoxia does not affect the levels of HLA-G in trophoblasts
P. Moreau et al.	1999	Clinical	Placenta	NB, RNAse Protection Assays, RT PCR	Inflammation			IL-10 induces HLA-G expression on trophoblasts and monocytes
S. Kshirsagar et al.	2012	Clinical and in vitro	Placenta	IF, WB	Immune Mechanism	1st trimester placental exosome	Cytotrophoblast derived exosomes are a source of exosome associated HLA-G5	
G. Chumbley et al.	1993	Clinical	Placenta	NB, SB	HLA-G Expression	Cytotrophoblast cell columns, interstitial trophoblast, endovascular trophoblast, placental bed giant cells, villous cytotrophoblast, villous mesenchymal cells. Not syncytiotrophoblast		
M. Stout et al.	2015	Clinical	Placenta	IHC	PTB/PPROM/Abortion		Higher levels of HLA-G associated with PTB	
L. Chen et al.	2010	In vitro (primary and immortalized cells)	PB, Placenta	FC, IHC, RT rPCR, WB	Immune Mechanism			HLA-G knockdowns diminished EVT cell resistance to NK mediated cytotoxicity
T. Poehlmann et al.	2006	In vitro (primary cells)	Placenta	WB	Immune Mechanism			sHLA-G reduced NK cell expression of STAT3 as well as several downstream effects, such as perforin expression, proliferation and cytotoxicity.
L. Lombardelli et al.	2016	In vitro (primary cells)	Decidua	FC, RT rPCR	Trophoblast Invasion			HLA-G5 is important for implantation
J. Nevalainen et al.	2017	Clinical	Placenta	Microarray	Preeclampsia			HLA-G mRNA increased in severe PE
D. Goldman-Wohl et al.	2000	Clinical	Placenta	IHC	Preeclampsia			Decreased HLA-G associated with PE
J. Lata et al.	1992	Clinical	Placenta	ISH	HLA-G Expression	1st trimester syncytiotrophoblast		
L. Marozio et al.	2017	Clinical	PB	ELISA	Preeclampsia	Peripheral blood	Low levels of maternal plasma sHLA-G associated with PE	
C. Mando et al.	2016	Clinical	CB, PB	dPCR	Preeclampsia	Peripheral blood	HLA-G 14 bp polymorphism unrelated to pregnancy hypertensive disorders or fetal growth restriction	
A. Lin et al.	2006	Clinical	Placenta	dPCR	Preeclampsia		HLA-G polymorphism not associated with PE	
I. Biyik et al.	2014	Clinical	PB	ELISA	PTB/PPROM/Abortion	Peripheral blood	First trimester maternal serum sHLA-G not correlated with high risk pregnancies	
S. Luo et al.	2017	In vitro (immortalized cells)	CL (HTR-8/Svneo)	FC, RT rPCR, WB	Preeclampsia		Decreased miR-148a in PE	miR-148a upregulates HLA-G post transcriptionally
L. Gorvel et al.	2014	In vitro (primary cells)	PB, Placenta	FC, IHC, Microarray, WB	Inflammation		Decidual DC express HLA-G and do not produce inflammatory cytokines after stimulation with LPS or peptidoglycan	
N. Sageshima et al.	2003	Clinical	Placenta	EM, IHC	Preeclampsia		Subset of extravillous trophoblasts that do not express HLA-G were found to also show signs of necrosis in both normal and PE placentas	
R. McIntire et al.	2008	Clinical	Placenta	IHC, RT rPCR	HLA-G Expression			The HLA-G receptors LILRB1 were found in placental stromal cells and LILRB2 in vascular smooth muscle

A. Orozco et al.	2009	Clinical and in vitro	CL (JEG-3), PB	FC	Preeclampsia	Placental microparticles		Peripheral blood DNA microparticles were associated with HLA-G, these levels are increased in 1st and 2nd trimester, as well as in term PE
G. Datema et al.	2003	Clinical	Fetal membranes, Placenta	IHC	Preeclampsia		No difference in HLA-G in chorionic or extravillous trophoblasts in preterm or PE patients, elevated HLA-G in term amnion from preterm deliveries	
S. Yie et al.	2006	In vitro (primary and immortalized cells)	CL (JEG, BEWO), Placenta	ELISA, RT rPCR, WB	Endocrine Interaction	First trimester trophoblasts and JEG-3		Progesterone upregulates HLA-G
S. Yie et al.	2006	In vitro (immortalized cells)	CL (JEG-3)	ELISA, EMSA	Endocrine Interaction			HLA-G regulation involves progesterone receptor element in HLA-G promoter region
A. Ishitani et al.	2003	Clinical	Placenta	ELISA, ICC, IHC, WB, Proteomics	HLA-G Expression	Extravillous trophoblasts (membrane bound), Placental trophoblasts (soluble)		
Y. Hamai et al.	1999	Clinical	Amniotic fluid	ELISA	HLA-G Expression	Amniotic fluid		
G. Chumbley et al.	1994	In vitro (primary cells)	Placenta	NB, WB	Immune Mechanism		HLA-G transfected cells resistant to decidual NK lysis	
C. Solier et al.	2002	In vitro (primary and immortalized cells)	CL (JAR, JEG-3), Placenta	ELISA, FC, IHC, RT rRNA, Sequencing, WB	Immune Mechanism	Placental villous trophoblast		sHLA-G1 from villous trophoblasts induced apoptosis of CD8+ cells
M. Han et al.	2014	In vitro (primary cells)	CL (BEWO), Decidua, Placenta	ELISA, RT rPCR, WB	Infection			Neutralizing HLA-G decreased dendric NK apoptosis in Toxoplasma gondii infection
F. Beneventi et al.	2016	Clinical	PB, Vaginal fluid	ELISA	PTB/PPROM/Abortion	Maternal serum, vaginal fluid	Elevated sHLA-G in peripheral blood and vaginal secretions of patients with PPROM	
W. Chu et al.	1998	In vitro (primary and immortalized cells)	CL (JAR, JEG-3), Placenta	ELISA, IHC, RT rPCR	Inflammation	Placental trophoblast, activated placental macrophages		
A. Lindaman et al.	2006	In vitro (immortalized cells)	CL (immortalized NK cells)	FC, IP	Immune Mechanism			sHLA-G effectively blocked NK lysis of target cells and induced peripheral NK cell apoptosis
J. McCormick et al.	2009	In vitro (immortalized cells)	CL (SGHPL-4)	ELISA, RT rPCR, WB	Trophoblast Invasion		sHLA-G may be involved in regulating trophoblast invasion	
Y. Guo et al.	2013	In vitro (primary and immortalized cells)	CL (JEG-3), Placenta	ELISA, IHC, RT rPCR, WB	Trophoblast Invasion		HLA-G5 induces trophoblast invasion via KIR2DL4 and LILRB1	
R. Rizzo et al.	2016	Clinical	CB, PB, Amniotic fluid	ELISA, WB	Infection			Elevated sHLA-G found in maternal peripheral blood and amniotic fluid of patients infected with CMV
M. Zdravkovic et al.	1999	In vitro (immortalized cells)	CL (JAR, JEG-3, HT-116, HTR-8)	IHC, RT rPCR, Sequencing, WB	Immune Mechanism		Cell lines expressing HLA-G are resistant to NK lysis	
P. Morales et al.	2007	In vitro (primary cells)	CL (HELA), Placenta	WB	HLA-G Expression		Villous cytotrophoblasts produce sHLA-G	
L. Rieger et al.	2002	In vitro (primary and immortalized cells)	CL (k-562, NKL)	ELISA	Immune Mechanism		HLA-G has immunosuppressive effects on large granular lymphocytes	
F. Eskicioglu et al.	2016	Clinical	Placenta	WB	PTB/PPROM/Abortion		HLA-G levels not associated with recurrent miscarriages	
D. Darmochwal-Kolarz et al.	2012	Clinical	PB	ELISA	Preeclampsia		Peripheral sHLA-G are highest in 2nd trimester (2nd>1st>3rd), sHLA-G in peripheral blood lower in PE	
A. Iversen et al.	2008	Clinical	CB, Placenta	dPCR, Sequencing, RT rPCR	Preeclampsia		14bp HLA-G polymorphism not associated with PE	
T. Tilburgs et al.	2015	In vitro (primary cells)	CL (primary fetal HLA-G, EVT), Decidua, PB, Placenta	Confocal, FC, RT dPCR, FC, WB	Immune Mechanism			dNK interaction with EVT leads to acquisition of membrane HLA-G of dNK cells via trogocytosis
J. Houlihan et al.	1995	Clinical	Fetal membranes	FC, IHC, Proteomics, RT rPCR	HLA-G Expression	Fetal membrane amnion and chorion		
A. Blaschitz et al.	2005	Clinical	Placenta	ELISA, IHC, WB	HLA-G Expression	Placental EVT		
X. Xu et al.	2013	In vitro and in vivo	Endometrium, Placenta	FC, RT rPCR	Infection			Toxoplasmosis in pregnancy elevates HLA-G
I. Manaster et al.	2012	Clinical and in vitro	CL (RKO, 721.221), PB, Placenta	IHC, Luciferase	HLA-G Expression			miR-148a and miR-152 down regulate HLA-G

Abbreviations	(EVT=extravillous trophoblast) (PTB=preterm birth, PE=preeclampsia)	(Chorionic villus sampline = CVS, EVT=extravillous trophoblast, CB=cord blood, CL= Cell line, PB=Peripheral Blood)	(IHC=immunohistochemistry, ICC=immunocytochemistry,FC=flow cytometry, WB=western blot, ELISA=enzyme linked immunoabsorbance assay, PCR=polymerase chain reaction, ISH=in situ hybridization, NB=northern blot, RT PCR=real time PCR, dPCR=DNA PCR, rPCR=RNA PCR, IF=immunoflouresence, IP=immunoprecipitation, SB=Southern Blot, EM=electron microscopy, EMSA=Electrophoretic mobility shift assay)	(PTB=preterm birth,PPROM=preterm premature rupture of membranes)
